# Synthesis, Molecular Docking Analysis, and Evaluation of Antibacterial and Antioxidant Properties of Stilbenes and Pinacol of Quinolines

**DOI:** 10.1155/2021/6635270

**Published:** 2021-03-02

**Authors:** Zeleke Digafie, Yadessa Melaku, Zerihun Belay, Rajalakshmanan Eswaramoorthy

**Affiliations:** ^1^Department of Applied Chemistry, Adama Science and Technology University, Adama, Ethiopia; ^2^Department of Applied Biology, Adama Science and Technology University, Adama, Ethiopia

## Abstract

Emergence of antimicrobial resistance to standard commercial drugs has become a critical public health concern worldwide. Hence, novel antimicrobials with improved biological activities are urgently needed. In this regard, a series of quinoline-stilbene derivatives were synthesized from substituted quinoline and benzyltriphenylphosphonium chloride using Wittig reaction. Furthermore, a novel pinacol of quinoline was synthesized by pinacolinazation of 2-methoxyquinoline-3-carbaldehyde which was achieved by aluminum powder-potassium hydroxide reagent combination at ambient temperature in methanol. The structures of the synthesized compounds were established based on their spectral data. The antibacterial activities of the synthesized compounds were evaluated in vitro by the paper disc diffusion method against two Gram-positive bacteria (*Staphylococcus aureus* and *Bacillus subtilis*) and two Gram-negative bacteria (*Escherichia coli* and *Salmonella typhimurium*). The best activity was displayed by compound **19** against E. coli with an inhibition zone of 16.0 ± 0.82 mm and 14.67 ± 0.94 mm at 500 and 250 *μ*g/mL, respectively. This is close to ciprofloxacin which is used as a positive control. The results of in silico molecular docking evaluation of the compounds against *E. coli* DNA gyraseB were in good agreement with the in vitro antibacterial analysis. Compounds 19 (−6.9 kcal/mol) and **24** (−7.1 kcal/mol) showed the maximum binding affinity close to ciprofloxacin (−7.3 kcal/mol) used as positive control. Therefore, the antibacterial activity displayed by these compounds is encouraging for further investigation to improve the activities of quinoline-stilbenes by incorporating various bioisosteric groups in one or more positions of the phenyl nuclei for their potential pharmacological use. Findings of the DPPH radical scavenging assay indicated that some of the quinolone stilbenes and pinacol possess moderate antioxidant properties compared to ascorbic acid used as a natural antioxidant.

## 1. Introduction

Treatment of infections caused by a variety of microbes including bacteria, viruses, and fungi has been a serious challenge. The problem is exacerbated by the emergence of multidrug microbial resistance to standard commercial drugs. The World Health Organization (WHO) has considered this antimicrobial drug resistance and the diminishing number of active antimicrobial drugs to be one of the greatest threats to human health. Moreover, problems of multidrug resistance of bacteria and fungi such as methicillin-resistant *Staphylococcus aureus* (MRSA), vancomycin- resistant *Staphylococcus aureus* (VRSA), vancomycin-resistant *enterococcus* (VRE), and fluconazol-resistant Candida have become a serious medical problem worldwide [1–3]. Hence, there is an urgent need to search for new effective antimicrobial agents with low side effects. The situation is even more significant and urgent; besides that mentioned above, opportunistic bacterial and/or fungal infections also endanger the health of a number of immune-weakened patients such as patients with AIDS, cancer, and transplants [4].

Quinoline moiety is one of the most important heterocyclic scaffolds present in many classes of biologically active compounds [5]. The quinoline nuclei, known since 1962, displays remarkable pharmacological properties such as antiplasmodium [6], anticancer [7–9], antibiotic [4, 10], antioxidant [4, 11], antimicrobial [12, 13], and antiinflamation [13]. Among quinolones, fluoroquinolones were known for their broad-spectrum antimicrobial activities and structural modifications that have resulted in the development of the first- to fifth-generation fluoroquinolone drugs [14, 15].

**Figure 1:**
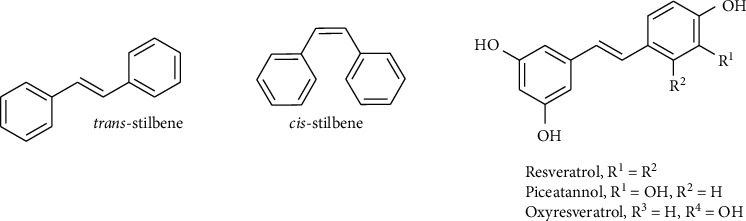
Chemical structures of some stilbenes.

Stilbenes are another class of biologically active compounds existing in two isomeric forms: (E)- stilbene (*trans*-stilbene) and (Z)- stilbene (*cis*-stilbene) (Figure 1) [16]. Resveratrol, piceatannol, and oxyresveratrol are recognized to be representatives of *trans*-polyphenolic stilbenes (Figure 1). Stilbene and its derivatives attracted much attention due to their various biological activities including anticancer, antiviral, antidiabetic, and antimicrobial activity and valuable for patients with cardiovascular diseases, dementia, and central nervous system (CNS) disorders [17–20].

Several studies have shown that the introduction of different functional groups into quinolines and stilbenes can improve their biological activities. Thus, due to their useful applications in various fields, scientists pay much attention to the modification of quinolines and stilbenes. Especially noteworthy in this regard is the emergence of new drugs with hybrid molecular architectures possessing better pharmacological properties than small molecules. Hence, in the present work, the synthesis of quinoline-stilbene conjugates and pinacols of quinoline was reported. The result of the synthesis of novel pinacols of quinoline was also reported here for the first time. Herein, we also report the results of the antibacterial and radical scavenging activities of synthetic derivatives of quinoline-stilbenes and pinacols of quinoline. It is rational to anticipate better bioactivity from a bigger scaffold developed by conjugating the phenyl ring to the quinoline nucleus which has wider sites for suspending different bioisosteres.

## 2. Materials and Methods

### 2.1. General

All solvents and organic reagents were purchased from LOVA CHEMIE PVT LTD. Melting points were determined using capillary tubes with the Japson analytical melting point apparatus and are uncorrected. The NMR spectra of the compounds were obtained using the NMR Bruker Avance 400 spectrometer operating at 400 MHz using DMSO-d6 and CDCl_3_ as a solvent, and chemical shifts (*δ*) were reported in ppm and the coupling constants (*J*) are reported in Hertz. The infrared spectra of the compounds were recorded using KBr pellets on a PerkinElmer BX IR Spectrometer (400–4000 cm^−1^) at Addis Ababa University. UV-Vis spectra were determined using a double beam UV-VIS Spectrophotometer (SM-1600 Spectrophotometer)) using methanol as a solvent. Analytical thin-layer chromatography was conducted on a 0.2 mm-thick layer of silicagel GF254 (Merck) on an aluminum plate, and the spots were visualized with 254 nm and 366 nm wavelength UV light. Silica gel gravity column chromatography was carried out using 100-mesh silica gel.

### 2.2. Synthesis

2-Chloroquinoline-3-carbaldehyde and 2-chloro-8-methylquinoline-3-carbaldehyde were prepared according to the literature report [21]. The various 2-substituted quinoline-3- carbaldehyde intermediates that were used in the present work were prepared by the methods that our research team reported previously [22]. The synthesized compounds and the synthetic procure used for each synthesis are summarized in the following tables (Tables 1–6).

The structures of the synthetic compounds synthesized by the procedure have been established using spectroscopic methods including UV-Vis, FT-IR, and NMR, and the result is summarized below.

#### 2.2.1. The Spectroscopic Data of 2-Chloroquinoline-3-carbaldehyde (4)

The crude product of 4 was purified by recrystallization from ethyl acetate. It was yellow crystal; mp 146–148°C; yield 49%; *R*_*f*_ = 0.22 (*n*-hexane : EtOAc = 9 : 1). UV-Vis (MeOH) *λ*_max_ = 280 nm; IR (ʋ cm^−1^, KBr): 3035 (CH-arom.), 1693 (C=O aldehyde), 1621 (quinoline C=N str.), and 599 (aromatic C=C str.); ^1^H NMR (400 MHz, CDCl_3_): *δ*H 7.65 (1H, *m*,H-6), 7.88 (1H, m, H-7), 7.97 (1H, *d*, *J* = 8.25 Hz, H-8), 8.07 (1H, *d*, *J* = 8.25 Hz, H-5), 8.74 (1H, *s*, H-4), and 10.54 (1H, s, H-9); ^13^C NMR (100 MHz, CDCl_3_): *δ*C 126.3 (C-1), 126.5 (C-8), 128.2 (C-6), 128.6 (C-4a), 129.7 (C-5), 133.6 (C-7), 140.3 (C-4), 149.6 (C-8a), 150.1 (C-2), and 189.1 (C-9).

#### 2.2.2. The Spectroscopic Data of (Z)-2-chloro-3-styrylquinoline (5)

The crude product of 5 was purified by silica gel column chromatography using n-hexane : acetate (9 : 1) as an eluent. The yield was 35.5%, a white powder; mp 58–60°C; *R*_*f*_ = 0.63 (*n*-hexane : EtOAc = 9 : 1). UV-Vis *λ*_max_ (MeOH) = 375 nm; IR (ʋ cm^−1^, KBr): 3035 (CH-arom.), 1635 (C=C alkene), 1621 (quinoline C=N str.), and 599 (aromatic C=C str.; ^1^H NMR (400 MHz, DMSO-d6): *δ*H 6.72 (1H, *d*, *J* = 12.25 Hz, H-10), 6.91 (1H, *d*, *J* = 12.25 Hz, H-9), 7.16 (2H, *m*, H-4′, H-5′), 7.44 (2H, *m*, H-6, H-7), 7.56 (1H, *td*, H-3′), 7.66 (1H, *m*, H-2′), 7.78 (2H, *m*, H-5′,H-6′), 7.94 (1H, *t, J* = 7.45 Hz, H-8), and 8.14 (1H, s, H-4); ^13^C NMR (100 MHz, DMSO-d6): *δ*C 125.7 (C-9), 127.2 (C-4), 127.4 (C-5), 127.9 (C-6), 128.0 (C-3), 128.3 (C-4′), 128.4 (C-8), 129.0 (C-2′, C-6′), 129.1 (C-3′, C-5′), 131.2 (C-10), 133.7 (C-4), 135.9 (C-7), 138.9 (C-11), 146.6 (C-2), and 149.9 (C-8a).

#### 2.2.3. The Spectroscopic Data of (Z)-3-styrylquinolin-2(1H)-one (7)

The crude product of 7 was purified by silica gel column chromatography using *n*-hexane : ethyl acetate (8 : 1) as an eluent. The yield was 40.5%, a dull yellow powder; mp 178–180°C; *R*_*f*_ = 0.29 (*n*-hexane : EtOAc = 6 : 1). UV-Vis *λ*_max_ (MeOH) = 400 nm; IR (ʋ cm^−1^, KBr): 3429 (N-H str.), 3023 (aromatic C-H), 1673 (alkene C=C str.), 1610 (quinoline C=N str.), and 1558 and 1465 (aromatic C=C str.); ^1^H NMR (400 MHz, DMSO-d6): *δ*H 6.56 (1H, *d*, *J* = 12.55 Hz,H-9), 6.75 (1H, *d*, *J* = 12.55 Hz, H-10), 7.09 (1H, *t*, *J* = 7.07 Hz, H-6), 7.23 (1H, *d*, *J* = 7.05 Hz,H-7), 7.32 (5H, *m*, H-2′, H-3′, H-4′, H-5′, H-6′), 7.44 (1H, *d*, *J* = 6.64 Hz, H-5), 7.59 (1H, *d*, *J* = 7.3 Hz, H-8). 7.66 (1H, s, H-4), and 11.97 (1H, s, H-1); ^13^C NMR (100 MHz, DMSO-d6): *δ*C 115.3 (C-8), 119.3 (C-4a), 122.4 (C-3), 125.4 (C-4), 127.0 (C-6), 127.9 (C-9), 128.9 (C-2′, C-6′), 129.0 (C-3′, C-5′), 129.3 (C-4′), 130.6 (C-7), 132.2 (C-5), 136.9 (C-1′), 137.5 (C-10), 138.7 (C-8a), and 161.8 (C-2).

#### 2.2.4. The Spectroscopic Data of 2-Methoxyquinoline-3-carbaldehyde (8)

Compound 8 was a gray powder; the yield was 90%; mp 106–108°C; *R*_*f*_ = 0.32 (n-hexane : EtOAc = 9 : 1). UV-Vis *λ*_max_ (MeOH) = 295 nm; IR (ʋ cm^−1^, KBr): 3065.4 (aromatic C-H str.), 2919.3 (aliphatic C-H str.), 2847 (aliphatic C-H str.), 1673 (aldehyde C=O str.), 1620 (quinoline C=N str.), and 1599 and 1579 (aromatic C=C str.); 1H NMR (400 MHz, CDCl_3_): *δ*H 4.22 (3H, *s*, H-10), 7.45 (1H, *t*, *J* = 7.3 Hz, H-7), 7.76 (1H, *t*, *J* = 7.7 Hz, H-6), 7.85 (2H, *m*, H-5, H-8), 8.60 (1H, *s*, H-4), and 10.49 (1H, *s*, H-9); ^13^C NMR (100 MHz, CDCl_3_): *δ*C 55.9 (C-10), 120.0 (C-3), 124.4 (C-6), 125.1 (C-4a), 127.1 (C-8), 129.8 (C-5), 132.6 (C-7), 140.0 (C-3), 149.0 (C-8a), 161.2 (C-2), and 189.4 (C-9).

#### 2.2.5. The Spectroscopic Data of (Z)-2-methoxy-3-styrylquinoline (9)

The crude product of 9 was purified by silica gel column chromatography using *n*-hexane : ethylacetate (9 : 1) as an eluent. The yield was 28.3%; it was a gray powder; mp 138–140°C; *R*_*f*_ = 0.52 (*n*-hexane : EtOAc = 9 : 1). UV-Vis *λ*_max_ (MeOH) = 322 nm; IR (ʋ cm^−1^, KBr): 3065.4 (aromatic C-H str.), 2919.3 (aliphatic C-H str.), 2847 (aliphatic C-H str.), 1630 (alkene C=C str.), 1620 (quinoline C=N str.), and 1599 and 1579 (aromatic C=C str.); ^1^H NMR (400 MHz, CDCl_3_): *δ*H 3.97 (3H, *s*, -OCH_3_), 6.65 (1H, *d*, *J* = 11.96 Hz, H-10), 6.78 (1H, *d*, *J* = 11.96 Hz, H-9), 7.21 (4H, *m*, H-3, H-5′, H-4′, H-6), 7.34 (1H, *m*, H-6′), 7.42 (1H, *m*, H-2′), 7.61 (2H, *m*, H-7, H-8), 7.78 (1H, *d*, H-5), and 7.91 (1H, *s,* H-4); ^13^C NMR (100 MHz, CDCl_3_): *δ*C 54.1 (-OCH_3_), 122.1 (C-3), 124.6 (C-4a), 124.7 (C-9), 126.9 (C-2′), 127.1 (C-8), 127.9 (C-5), 128.0 (C-6′), 128.8 (C-6, C-5′, C-3′), 129.29 (C-4′), 130.0 (C-10), 132.7 (C-4), 136.7 (C-7), 137.7 (C-1′), 145.6 (C-8a), and 160.2 (C-2).

#### 2.2.6. The Spectroscopic Data of 2-(((2-((2-hydroxyethyl)amino)quinolin-3-yl)methylene)amino)ethan-1-ol (10)

Compound 10 was a yellow powder, and the overall yield was 84%; mp 80–82°C; *R*_*f*_ = 0.25 (*n*-hexane : Methanol = 7 : 3). UV-Vis *λ*_max_ (MeOH) = 390 nm; IR (ʋ cm^−1^, KBr): 3525–3510 (O-H and N-H), 3065.4 (aromatic C-H str.), 2919.3 (aliphatic C-H str.), 2847 (aliphatic C-H str.), 1623 (imine C=N str.), 1620 (quinoline C=N str.), and 1599 and 1579 (aromatic C=C str.) ^1^H NMR (400 MHz, DMSO-d6): *δ*H 3.65 (8H, *d*, H-13, H-17, H-11, H-15), 4.72 ((1H, *s*, H-12), 4.92 (1H, *s*, H-12), 7.19 (1H, *t*, *J* = 7.25 Hz, H-6), 7.55 (2H, *m*, H-5, H-8), 7.72 (1H, *d*, *J* = 8.36 Hz, H-7), 8.21 (1H, *s*, H-4), 8.5 (1H, *s*, H-9), and 9.55 (1H, *s*, NH); ^13^C NMR (100 MHz, DMSO-d6): *δ*C 43.4 (C-14), 60.5 (C-12), 61.2 (C-15), 63.7 (C-11), 117.2 (C-3), 121.9 (C-8), 122.4 (C-4a), 125.7 (C-5), 128.9 (C-6), 131.5 (C-7), 143.0 (C-4), 148.3 (C-8a), 155.4 (C-2), and 163.8 (C-9); Dept-135 *δ*C 43.4 (C-14 down), 60.5 (C-12 down), 61.2 (C-15 down), 63.7 ( C-11 down), 121.9 (C-8), 125.7 (C-5), 128.9 (C-6), 131.5 (C-7), 143.0 (C-4), and 163.8 (C-9).

#### 2.2.7. The Spectroscopic Data of 2-Chloro-8-methylquinoline-3-carbaldehyde (13)

The crude product 14 was recrystallized from EtOAc. Yield after recrystallization was 38.75%, pale yellow crystal; mp 139 140°C; *R*_*f*_ = 0.30 (*n*-hexane : EtOAc = 9 : 1). UV-Vis *λ*_max_ (MeOH) = 295 nm; IR (ʋ cm^−1^, KBr) 3223, 3025 (arom-C-H.), 2956 (alip-C-H), 2837 (alip-C-H), 1683.3 (C=O str.), 1621 (quinoline C=N str.), and 1579 (aromatic C=C str.); ^1^H NMR (400 MHz; CDCl_3_): *δ*H 2.80 (3H, *s*, H-10), 7.53 (1H, *t*, *J* = 7.78 Hz, H-6), 7.70 (1H, d, H-7), 7.78 (1H, d, *J* = 7.45 Hz, H-5), 8.70 (1H, s, H-4), and 10.6 (1H, s, H-9); ^13^C NMR (100 MHz, CDCl_3_): *δ*C 17.8 (C-10), 126.0 (C-3), 126.5 (C-6), 127.5 (C-5), 127.8 (C-4a), 133.6 (C-7), 136.9 (C-8), 140.4 (C-4), 148.7 (C-2), 149.4 (C-8a), and 189.5 (C-1).

#### 2.2.8. The Spectroscopic Data of (Z)-2-chloro-8-methyl-3-styrylquinoline (14)

The crude product of 14 was purified by silica gel column chromatography (solvent *n*-hexane : ethyl acetate 9 : 1). The yield was a yellow gum; *R*_*f*_ = 0.7 (*n*-hexane : EtOAc = 9 : 1). UV-Vis *λ*_max_ (MeOH) = 375 nm; IR (ʋ cm^−1^, KBr) 3025 (arom-C-H.), 2956 (alip-C-H), 2837 (alip-C-H), 1630 (alkene C=C str.), 1621 (quinoline C=N str.), and 1579 (aromatic C=C str.); ^1^H NMR (400 MHz, DMSO-d6): *δ*H 7.36 (1H, *m*, H-4′), 7.44 (4H, *m*, H-5′, H-3′, H-9, H-10), 7.52 (H, *dd*, H-6′), 7.61 (1H, d*t*, H-2′), 7.66 (2H, *m*, H-6, H-7), 7.85 (1H, *dd*, H-5), and 7 and 8.78 (1H, s, H-4); ^13^C NMR (100 MHz, DMSO-d6): *δ*C 17.5 (C-11), 123.1 (C-6), 126.4 (C-6), 126.4 (C-9), 127.4 (C-5, C-8), 127.7 (C-9), 127.8 (C-4′), 128.1 (C-6′), 129.4 (C-3′, C-5′), 129.7 (C-4a), 131.1 (C-2′), 133.9 (C-7), 135.4 (C-4), 135.8 (C-8), 136.8 (C-1′), 145.7 (C-2), and 148.7 (C-8a).

#### 2.2.9. The Spectroscopic Data of 8-methyl-2-oxo-1, 2-dihydroquinoline-3-carbaldehyde (15)

Compound 15 was yellow powder; mp 176–178°C; *R*_*f*_ = 0.41 (n-hexane : EtOAc = 1 : 1). UV-Vis *λ*_max_ (MeOH) = 375 nm, IR (ʋ cm^−1^, KBr) 3429 (O-H str.), 3179.5 (NH str.), 3023 (aromatic C-H), 2919.8 (aliphatic CH-str'.), 2837.2 (aliphatic C-H) 1673 (C=O str.), 1610 (quinoline C=N str.), and 1558 and 1465 (aromatic C=C str.); ^1^H NMR (400 MHz; CDCl_3_): *δ*H 2.56 (3H, s, C-10), 7.23 (1H, *t*, *J* = 7.5 Hz, H-6), 7.51 (1H, *d*, *J* = 7.32 Hz, H-7), 7.61 (1H, *m*, *J* = 7.7 Hz, H-5), 8.49 (1H, *s*, H-4), and 10.48 (1H, s, H-9); ^13^C NMR (100 MHz, CDCl_3_): *δ*C 16.7 (C-10), 117.9 (C-6), 123.3 (C-3, C-8), 129.1 (C-4a, C-7), 135.0 (C-5), 139.1 (C-8a), 144.2 (C-4), 162.7 (C-2), 190.00 (C-9).

#### 2.2.10. The Spectroscopic Data of (Z)-8-methyl-3-styrylquinolin-2(1H)-one (16)

The crude product of **16** was purified by column chromatography with solvent *n*-hexane: ethylacetate (8 : 1). The pure product was a dull yellow powder; mp 60–62°C; *R*_*f*_ = 0.38 (*n*-hexane : EtOAc = 6 : 1).

UV-Vis *λ*_max_ (MeOH) = 375 nm; IR (ʋ cm^−1^, KBr): 3429 (N-H str.), 3023 (aromatic C-H), 2919.8 (aliphatic CH-str'.), 2837.2 (aliphatic C-H), 1630 (alkene C=C str.), 1610 (quinoline C=N str.), and 1558 and 1465 (aromatic C=C str.); ^1^H NMR (400 MHz, DMSO-d6): *δ*H 2.56 (3H, *s*, C-11), 7.11 (1H, *t*, *J* = 8.46 Hz, H-6), 7.24–7.42 (5H, *m*, H-10, H-7, H-5, H-3′, H-5′), 7.69 (4H, *m*, H-2′, H-4′, H-6′, H-9), 8.23 (1H, *s*, H-4), and 11.10 (1H, *s*, H-1); ^13^C NMR (100 MHz, DMSO-d6): *δ*C 17.7 (C-11), 119.9 (C-8), 122.37 (C-6), 123.4 (C-4), 123.6 (C-5), 123.7 (C-9), 126.5 (C-4′), 127.0 (C-2′, C-6′), 128.3 (C-4a), 128.4 (C-3′, C-5′), 131.3 (C-7), 133.7 (C-3), 136.1 (C-10), 137.7 (C-1′), 136.7 (C-8a), and 162.1 (C-2).

#### 2.2.11. The Spectroscopic Data of 8-Methyl-N-(4-nitrophenyl)-3-styrylquinolin-2-amine (19)

The crude product of 19 was purified by silica gel column chromatography using *n*-hexane : ethylacetate (7 : 1). The pure product was a pink powder; mp 150–152°C; *R*_*f*_ = 0.61 (*n*-hexane : EtOAc = 7 : 3). UV-Vis *λ*_max_ (MeOH) 315 nm, 319 nm; IR (ʋ cm^−1^, KBr): 3055 (aromatic C-H str.), 1620 (alkene C=C str.), 1615 (quinoline C=N str.), 1577 (aromatic C=C str.), and 1512 and 1340 (O=N-O str.); ^1^H NMR (400 MHz, DMSO-d6): *δ*H 2.36 (3H, *s*, -CH_3_), 6.78 (1H, *d*, *J* = 12.75 Hz, H-10), 6.88 (1H, *d*, *J* = 12.75 Hz, H-9), 7-21-7.78 (22H, *m*, H-5, H-6, H-7, H-9, H-12, H-13, H-14, H-15, H-16, H-18, H-22 and their stereoisomers), 8.14 (1H, *d*, H-6′), 8.26 (2H, *m*, H-19 and its stereoisomer), 8.33 (2H, *m*, H-21 and its stereoisomers), and 8.72 (1H, *s*, NH), ^13^C NMR (100 MHz, DMSO-d6): *δ*C 17.4 (-CH_3_), 121.1 (C-9), 122.3 (C-18, C-21), 122.7 (C-6), 123.6 (C-5), 127.3 (C-19, C-22), 128.1 (C-14), 128.8 (C-12, C-16), 129.3 (C-13, C-15), 130.5 (C-10), 133.8 (C-7), 135.0 (C-3, C-4a), 136.9 (C-8, C-11), 137.1 (C-20), 140.0 (C-4), 143.6 (C-17), 157.2 (C-2), and 159.2 (C-8a), and peaks due to their stereoisomers were also seen.

#### 2.2.12. The Spectroscopic Data of (Z)-N-(4-((8-methyl-3-styrylquinolin-2-yl)methyl)phenyl)acetamide (22)

The crude product 22 was purified by silica gel column chromatography with n-hexane : ethyl acetate (7 : 4) as eluent. mp 178–180°C; *R*_*f*_ = 0.42 (n-hexane : EtOAc = 7 : 5). UV-Vis *λ*_max_ (MeOH); IR (ʋ cm^−1^, KBr) 3281.8 (N-H str), 3055 (aromatic C-H str.), 1645.7 (NHC=O) 1620 (alkene C=C str.), 1615 (quinoline C=N str.), and 1577 (aromatic C=C str.); ^1^H NMR (400 MHz, DMSO-d6): *δ*H 2.3 (3H, *s*, H-24), 2.33 (3H, *s*, 25), 6.81 (1H, *d*, *J* = 12.75 Hz, H-10), 6.89 (1H, *d*, *J* = 12.75 Hz, H-9), 7.08 (2H, *d*, *J* = 8.97 Hz,H-18, H-22), 7.25 (5H, *m*, H-6, H-13, H-14, H-15, H-16), 7.42 (1H, *m*, H-12), 7.63 (2H, *m*, H-21, H-19), 7.68 (1H, *d*, *J* = 8.97, H-5), 8.06 (1H, s, H-4), and 8.66 (NH); ^13^C NMR (100 MHz, DMSO-d6): *δ*C 17.4 (C-25), 24.4 (C-24), 120.0 (C-8, C-22), 122.3 (C-19, C-21), 124.2 (C-6), 125.1 (C-5), 125.5 (C-3, C-4a), 127.2 (C-9), 128.0 (C-28), 128.9 (C-12, C-16), 129.3 (C-13), 130.3 (C-15), 133.3 (C-7), 134.7 (C-20) 136.4 (C-8), 137.0 (C-11), 139.2 (C-10), 143.9 (C-17), 148.9 (C-8a), 158.6 (C-23), and 168.6 (C-2).

#### 2.2.13. The Spectroscopic Data of Methoxy-5-methylquinolin-3-yl)-2-(2-methoxy-8-methylquinolin-3-yl)ethane-1,2-diol (24)

Compound 24 was purified by silica gel column chromatography using solvent n-hexane : ethyl acetate (8 : 1). The final product was a white powder; mp 202–204°C; *R*_*f*_ = 0.4 (n-hexane : EtOAc = 7 : 3); UV-Vis *λ*_max_ (MeOH) 325 nm; IR (ʋ cm^−1^, KBr): 3470 (br-alcohol -CHO-H str.), 3013 (aromatic C-H str.), 2930.9 (aliphatic C-H str.), 1625 (quinoline C=N str.), 1620 (alkene C=C str.), and 1589.6 and 1475.5 (aromatic C=C str.); ^1^H NMR (400 MHz, DMSO-D_6_): *δ*_*H*_ 2.65 (3H, *s*, H-10), 3.99 (3H, *s*, H-11), 5.20 (1H, *d*, *J* = 7.51 Hz, H-9), 5.25 (1H, *d*, *J* = 7.51 Hz, H-12), 7.31 (1H, t, *J* = 8.13 Hz, H-6), 7.51 (H, d, *J* = 8.11 Hz, H-7), 7.72 (1H, *d*, *J* = 8.11 Hz, H-5), and 8.28 (1H, s, H-4); ^13^C NMR (100 MHz, DMSO-d6): *δ*_C_ 17.3 (C-10), 52.9 (C-11), 69.2 (C-9), 117.2 (C-6), 124.79 (C-3), 125.33 (C-5), 127.1 (C-4a), 128.9 (C-7), 133.8 (C-8), 136.7 (C-4), 143.6 (C-8a), and 158.26 (C-2).

### 2.3. Bioactivity

#### 2.3.1. Antibacterial Activity

Four strains of bacterial species, two Gram-positive bacteria (*Staphylococcus aureus (ATCC25923*) and *Bacillus subtilis* (ATCC6633)), and two Gram-negative bacteria (*Escherichia coli* (ATCC, 25922) and *Salmonella typhimurium* (ATCC 13311)) were obtained from the Adama Public Health Research & Referral Laboratory Center to evaluate the antibacterial activity of the synthetic compounds. The identity of the bacterial strains were recognized and confirmed by morphology of colony and Gram staining and by standard biochemical tests following the methods of Bergey's Manual of Determinative Bacteriology (1994) [23]. The bacterial strains were brought to the microbiology laboratory with nutrient agar and preserved at 4°C until they are used. The antibacterial efficacy of the compounds was tested by using the disc diffusion method. The microbial cultures were grown overnight at 37°C in nutrient broth, adjusted to 0.5 McFarland standard using distilled water, and lawn inoculated onto Mueller-Hinton agar (MHA) plates. The synthetic compounds were dissolved in DMSO and adjusted to a concentration of 250 and 500 *μ*g/mL. Sterile filter paper discs of 6 mm diameter were soaked in 1 mL DMSO solution of the compounds at 250 and 500 *μ*g/mL concentrations. Then, the saturated paper discs were placed on the centre of each MHA plate. Ciprofloxacin was the standard drug used as positive control, and DMSO was used as negative control. The plates were then inverted and incubated for 24 hours at 37°C, and the zone of inhibition was recorded. The results were expressed as the mean of three measurements (Table 7).

#### 2.3.2. DPPH Radical Scavenging Activity

DPPH radical scavenging activity was used to evaluate the antioxidant activity of the synthetic compounds and compared with ascorbic acid (Table 2). All synthetic compounds were separately dissolved in methanol and serially diluted using 0.004% methanolic solution of DPPH to furnish 12.5, 25, 50, and 100 *μ*g/mL. After incubating the mixtures at 37°C for 30 min, the absorbance was measured with a double-beam spectrophotometer (517 nm). The DPPH radical scavenging rate of each sample was calculated as follows [4, 22]:

(1) $$\begin{array}{c} \%\text{ inhibition}=\frac{\left(A_{o}-A_{1}\right)}{A_{o}}\times 100, \end{array}$$

where *A*_*o*_ is the absorbance of the control reaction and *A*_1_ is the absorbance in the presence of the test or standard sample.

**Figure 2:**
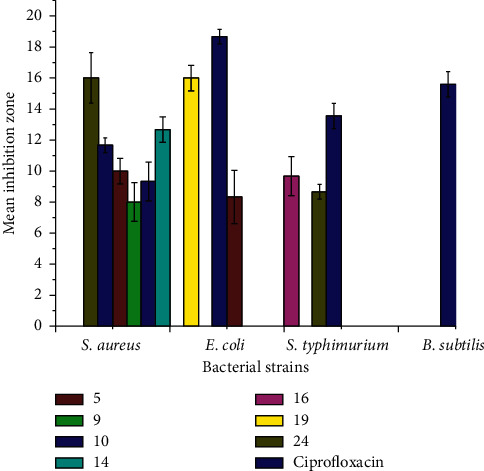
The binding interactions of ciprofloxacin against *E.coli* DNA gyrase A (PDB ID: 1ZI0).

The control DPPH solution was prepared by mixing 2 mL 0.004% DPPH with 2 mL methanol to afford 0.002% DPPH methanolic solution. The results were presented in percent radical scavenging activity (Table 8), and the same results have been demonstrated using a line graph (Figure 2).

#### 2.3.3. Molecular Docking Studies

To investigate the mode of interaction between the *E. coli* gyrase A and synthetic compounds in a 3D fashion, the compounds were docked within the binding site of the protein. AutoDock Vina with our previously reported protocol was used to dock the proteins (PDB ID: 1ZI0, PDB ID: 6F86, and PDB ID: 2XCT) and compounds (**5–24**) into the active site of proteins [22, 23]. The 2D chemical structures of the compounds were drawn using Chem Office tool (Chem Draw 16.0) assigned with proper orientation followed by the energy minimization of each molecule using ChemBio3D. The energy-minimized ligand molecules were then used as input for AutoDock Vina, to carry out the docking simulation [22, 23]. The crystal structure of the receptor molecules *E. coli* DNA gyrase A (PDB ID 1ZI0) and *S. aureus* Gyrase complex with ciprofloxacin and DNA (PDB ID: 2XCT) was downloaded from protein data bank. The protein preparation was carried out using the reported [24] standard protocol by removing the cocrystallized ligand, deleting water molecules, and adding polar hydrogens and cofactors, and then, the target protein file was prepared by leaving the associated residue with protein by using Auto Preparation of target protein file Auto Dock 4.2 (MGL tools1.5.6). The graphical user interface program was used to set the grid box for docking simulations. To surround the region of interest in the macromolecule, the grid was used. The best docked conformation between the compounds and the protein was explored with the docking algorithm provided with Auto Dock Vina [24]. During the docking process, a maximum of nine conformers were considered for each ligand. The conformations with the most favourable (least) free binding energy were selected for analyzing the interactions between the target receptor and ligands by Discovery suite visualizer. The ligands are represented in different colour, and H-bonds and the interacting residues are represented in stick model representation.

### 2.4. Statistical Analysis

Experiments were conducted in triplicates. The data presented are mean ± SD of the three independent experiments. GraphPad Prism version 5.00 for Windows was used to perform the Analysis (GraphPad Software, San Diego California USA, http://www.graphpad.com). Groups were analyzed for significant differences using a linear model of variance analysis (ANOVA) test for comparisons, with significance accepted for *p* < 0.05.

## 3. Results and Discussion

**Scheme 1:**
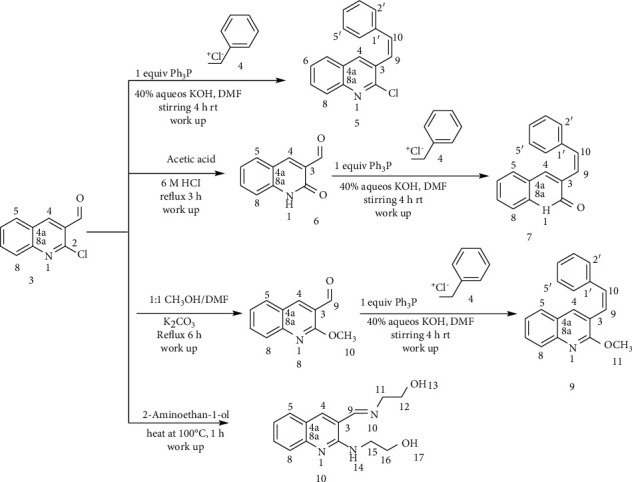
Synthesis of 2-chloroqunoinoline analog-stilbene.

**Scheme 2:**
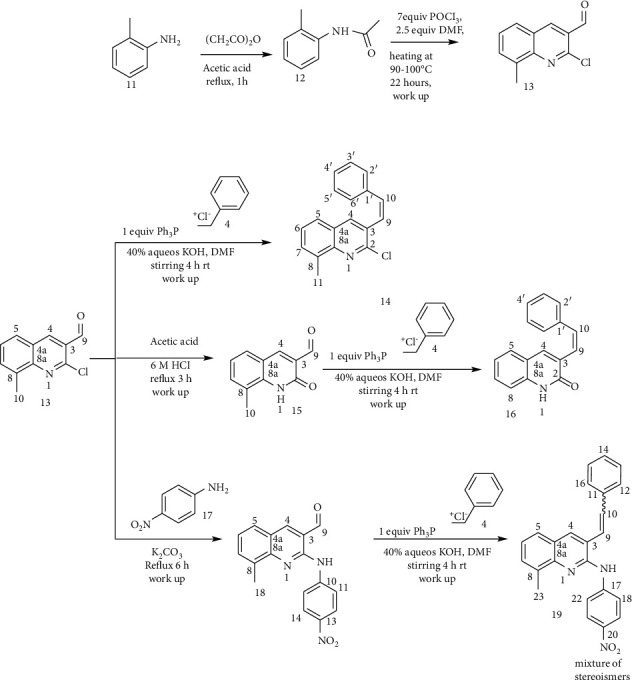
Synthesis of 2-chloro-8-methylquinoline-3-carbaldehyde analog-stilbenes.

The hybridization of biologically active molecules is a nice tool for drug development used to treat a variety of diseases. It provides a means to improve the bioactivity of bioactive molecules through synergetic effect. Hybrid drugs can provide combination therapies in a single multifunctional agent [6, 24, 25]. Based on this principle, in this paper, a series of a new hybrid of quinolone-stilbenes and pinacol of quinolines were synthesized and their biological activities were evaluated. In this paper, acetanilide (**1**) and 2-methylacetanilide (**11**) were synthesized by acetylation of aniline and *o*-toluidine in acetic anhydride-acetic acid mixtures (Schemes 1 and 2). Then, 2-chloroquinoline-carbaldehyde (**3**) and 2-chloro-8-methylquinoline-3-carbaldehyde (**13**) were developed by the application of Vilsmeier–Haack reaction (Schemes 1 and 2) using POCl_3_ in DMF [21, 24]. Subsequent replacements of chlorine by various nucleophiles were achieved using *N,N*-dimethyl formamide (DMF) as a solvent and K_2_CO_3_ as a base. The latter was selected due to its weak basicity and poor nucleophilic character which does not interfere with nucleophiles in most nucleophilic substitution reactions. The attractive features of DMF including high dielectric constant, its aprotic nature, wide liquid range, low volatility, dissolving all reactants, and supplying sufficient activation energy for the reactions allow us to use this reagent as a solvent. Furthermore, it is also miscible in water which eases the isolation of the desired product by adding the reaction mixture into cold ice water which causes the water-insoluble product to precipitate out [26]. Compounds 6 and 15 were prepared by refluxing 2-chloroquinoline-3-carbaldehyde and 2-chloro-methylquinoline-3-carbaldehyde in a mixture of acetic acid and 6 M aqueous hydrochloric acid. The desired products were obtained in excellent yields after removing acetic acid by distillation under reduced pressure. An attempt to displace the chlorine atom in compound 3 with 2-aminoethan-1-ol furnished undesired compound 10 in excellent yield (84%). This might be via a classical condensation reaction of the amine group of 2-aminethanol with the carbonyl of compound 3.

**Scheme 3:**
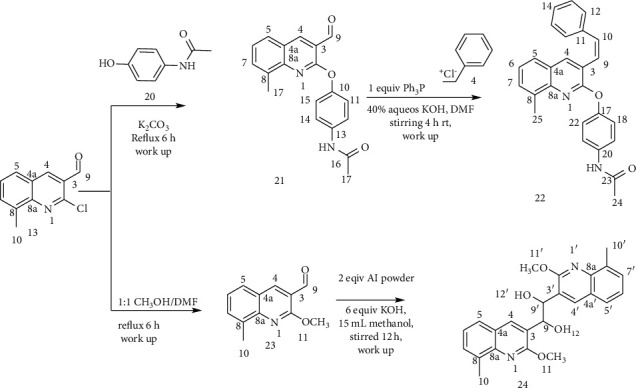
Synthesis of quinoline-stilbenes and quinoline pinacol.

After securing crucial intermediates 3 and 13, attention was given to the synthesis of a series of stilbenes quinolines and their substituted analogs using Wittig reaction. In this regard, the desired products were made by reacting derivatives of compounds 3 and 13 with benzyltriphenylphosphonium chloride using DMF as a solvent and KOH as a base. The products produced are mainly *cis* isomers which were confirmed by the coupling constant of the olefinic double bonds in the stilbenes. This agreed very well with previous products of Wittig reactions carried out at ambient temperature in DMF/KOH which gives the *cis*-stilbenes in good yield [27]. However, compound 19 was produced as a mixture of *cis* and *trans* stereoisomers. A further attempt to purify 19 using silica gel column chromatography was unsuccessful since the spots of the two stereoisomers overlapped in its TLC profile. Pinacolization of carbonyls has been conducted by using a number of reagents such as Mg-MgI_2_ [28], Zn-ZnC1_2,_ transition metals, actinides, and lanthanides [29]. Ti^II^ and Ti^III^ reagents have also received considerable attention, although olefination is a competing reaction with these reagents [29]. Among the various pinacolization agents of aromatic carbonyl compounds, the aluminium powder and potassium hydroxide in methanol were chosen and applied in the synthesis since they were reported to provide coupling rapidly [26–28]. In this reaction, a ratio of 1 : 2 : 6 equivalent of 2-methoxyquinoline-3-carbldehyde : aluminium : KOH was used in the process [30]. Methanol was chosen as a solvent because the reaction was fast and the side products were minimum in it [31] (Scheme 3). The crude product was purified by by silica gel column chromatography.

**Figure 3:**
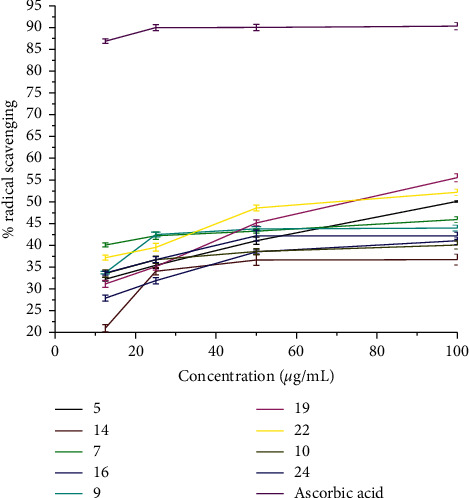
Antibacterial activity of synthetic compounds at 500 *μ*g/mL.

Quinolines and stilbenes and their analogs have been associated with various biological activities. Compounds containing the quinoline moiety have shown good amoebicidal, bactericidal, fungicidal, and antimalarial activities [29, 32]. Expecting similar trends, the antibacterial activities of the new synthetic hybrid compounds were screened against two Gram-negative and two Gram-positive bacteria (*S.aureus* (ATCC25923), *B.subtilis* (ATCC6633), *E. coli* (ATCC, 25922), and *S. typhimurium* (ATCC 13311)) using the paper disc diffusion method. The results of this evaluation are presented in Tables 7 and 8. It is further elaborated using a bar graph and depicted in Figure 3. The results in Table 8 show that some of the compounds exhibited good antibacterial activity. The mean inhibition zone for five of the synthetic compounds against *E. coli* varies from 8.0 ± 0.82 to 16.0 ± 0.82 mm, whereas for standard drug (ciprofloxacin) at the same concentration, it was 18.67 ± 0.47 diameter. Compounds 14 (with a mean inhibition zone of 12.67 ± 0.82 mm) and 19 (with a mean inhibition zone of 16.0 ± 0.82 mm) were the ones with maximum activity against *E. coli.*

*Z*-factor is a reflective of both the assay signal dynamic range and the data variation associated with signal measurements and, therefore, is suitable for assay quality assessment [33]. *Z*-factor provides a useful tool for comparison and evaluation of the quality of assay and can be utilized in assay optimization and validation [33]. The *Z*-factor is defined in terms of four parameters: the means (*μ*) and standard deviations (*σ*) of the sample and the positive and negative controls as shown below [30, 31].

(2) $$\begin{array}{c} Z=1-\frac{3\sigma_{s}+3\sigma_{c}}{\left|\mu_{s}-\mu_{c}\right|}, \end{array}$$

where *σ*_*s*_ and *σ*_*c*_ are the standard deviation of the sample and control and *μ*_*s*_ and *μ*_*c*_ are the mean of the sample and control.

A *Z*′-factor can be calculated using only control data as follows:

(3) $$\begin{array}{c} Z\prime =1-\frac{3\sigma_{c+}+3\sigma_{c-}}{\left|\mu_{c+}-\mu_{c-}\right|}, \end{array}$$

where *σ*_*c*+_ and *σ*_*c*−_ are the standard deviation of the positive and negative and *μ*_*c*+_ and *μ*_*c*−_ are the mean of the positive and negative control, respectively.

The *Z*-factor of the synthetic compounds and standards was calculated, and the results are presented in Table 7. The result of *Z*′-factor for the controls ranges from 0.82 to 0.92, a reflective of excellent quality assay. It was also found out that the *Z*-factor for six samples was greater than 0.5, while for another six samples, the *Z*-value was between 0.0 to 0.5. For four and two samples, the values were between −0.5 to 0 and between −0.5 to −1.0, respectively. Hence, the data obtained showed a good overall assay.

Compounds 5, 16, and 24 have moderate activity against *S*. *typhimurium*. Within the series, compound 16 with a mean inhibition zone of 9.67 ± 0.47 mm diameter was the maximum while a 13.56 ± 0.82 mm mean inhibition zone was observed for the standard drug at the same concentration. In addition, 24 showed the strongest activity against *S. aureus* with a 16.0 ± 1.63 mm mean inhibition zone compared to 11.67 ± 0.47 mm for the standard drug. However, none of the compounds showed activity against *B. subtilis.* Overall, among the compounds reported herein, only five of them displayed potent bioactivity.

In the current synthetic compound, the phenyl nucleus did not bear any substituents and the substituents in the quinoline nucleus were limited to the second and eight position. As a limitation, quinoline analogs synthesized by application of Vilsmeier–Hack reaction require activated aniline derivatives (activated *ortho* position relative to the amino group) to afford sufficient yields. However, most important bioisosters such as F, Cl, carbonyl, carboxylic acid, and sulfonyl group are electron withdrawers and deactivators and cannot be incorporated in the quinoline nucleus. But, the current hybrid scaffold overcomes this limitation since the phenyl nucleus can bear various types of functional groups (electron withdrawer or donors) in one or more of the five positions. Thus, the current strategy provides a manageable way to optimize the bioactivity through structural modification. Owing to Wittig reaction tolerance of various functional groups, phenyl ring can comprise one or more bioisosters which would be used to improve the bioactivity of quinoline-stilbenes. Regarding compound 24, which was generated by pinacolization of 2-chloro-8-methylquinoline-3-carbaldehyde, a huge improvement in the bacterial activity was observed relative to the monoalcohol (3-methoxy-5-methylnaphthalen-2- yl) methanol of the same reactant. Previously, we reported [22] (3-methoxy-5- methylnaphthalen-2-yl) methanol had poor antibacterial activity. It exhibited activity only against *E. coli* (with mean inhibition zone of 9.33 ± 0.89 mm diameter), whereas the correspondent diol (24) was active against both *S. aureus* (with a mean inhibition zone of 16.0 ± 1.63 mm diameter) and *S. typhimurium* (with a mean inhibition zone of 8.67 ± 0.47 mm diameter), respectively. Furthermore, the study revealed that there is an opportunity of developing stronger bioactive molecules by pinacolization of quionoline-3-carbaldehyde derivatives bearing various bioisosteric substituents.

The radical scavenging activities of the compounds (Table 9) showed that quinoline-stilbene hybrids were also having good antioxidant property. Antioxidants react with free radicals by different mechanisms—hydrogen atom transfer (HAT) or single-electron-transfer mechanism (SET) or the combination of both HAT and SET mechanisms [34]. The antioxidant activity of a compound depends on temperature and time [35]. Thus, the measurement of the antioxidant properties was carried out after keeping the sample-DPPH mixture in a dark incubator for 30 minutes at 37°C to achieve a stable equilibrium state before evaluation.

Most of the synthetic quinolone stilbenes and their analogs possess moderate antioxidant activity relative to ascorbic acid (Table 9). Compounds 5, 19, and 22 were only strong just as half of the ascorbic acid at the same concentration, and the others were even weaker. However, the procedure can be optimized to increase the antioxidant properties by introducing hydroxyl, thiol, and selenium groups on the phenyl nuclei as much as required. The IC_50_ values of the synthetic compounds are calculated and presented in Table 9. The lower the IC_50_, the higher the antioxidant activity of substances. IC_50_ values vary from 33.41 *μ*g/mL for 14 to 43.32 *μ*g/mL for 5. As observed from Table 9, the synthesized compounds displayed lower IC_50_ values compared to ascorbic acid (4.50 *μ*g/mL) used as a natural antioxidant. This indicates that the activities shown by the synthetic compounds are moderate in antioxidant activity. This is in good agreement with the literature reported for closely related compounds [11, 33].

The calculated *Z*-factors for the radical scavenging activity of the synthetic compounds lie between 0.88 and 0.99 (Table 10), while it was from 0.97 to 0.98 for ascorbic acid used as positive control. This clearly indicates the assay is an excellent assay and statistically accepted. Hence, the acceptable values of the *Z*-factor for the antibacterial and radical scavenging activities clearly indicate the acceptability of the bioassays.

### 3.1. In Silico Molecular Docking Evaluation

DNA gyrase is an enzyme belonging to a member of bacterial topoisomerase which controls the topology of DNA [35–37]. Hence, in this article, the molecular docking elucidation of the synthetic compounds was carried out to examine their binding pattern to *E. coli* DNA gyrase A and compared with standard clinical drug (ciprofloxacin). The compounds (5–24) were displayed a minimum binding energy ranging from −5.6 to −7.1 kcal/mol (Table 10), with the best result achieved using compounds 14 (−6.7 kcal/mol), 16 (−6.7 kcal/mol), 19 (−6.9 kcal/mol), and 24 (−7.1 kcal/mol. The binding affinity, H-bond, and residual interaction of the nine compounds and ciprofloxacin are presented in Table 11. All the compounds display interactions within the binding site of the clinical drug ciprofloxacin. The synthesized compounds (5–23) showed a residual interaction profile with amino-acid residues Ala-633, Val-685, Leu-735, Val-787, and Arg- 838, and H-bond showed that with Ile-683, Val-737, and Gln-788. Among the synthesized compounds, 23 (−7.1 kcal/mol) has resemblence in the hydrogen bonding and hydrophobic interactions of ciprofloxacin (−7.3 kcal/mole) within the binding cavity. The compounds 10 and 16 have shown hydrogen bond interaction with amino-acid residue Leu-735. The compounds **10** and **23** have shown hydrogen bond interaction with amino-acid residue Ile-683 similar to ciprofloxacin. Compounds 7 (Val-737), 14 (Arg-838, 19 (Arg-630, Thr-632), and 22 (Ile-634, Arg-580) have shown hydrogen bond interaction with different amino-acid residues. The in silico interaction results are agreeing with in vitro antimicrobial analysis of the synthesized compounds against *E.coli*.

**Figure 5:**
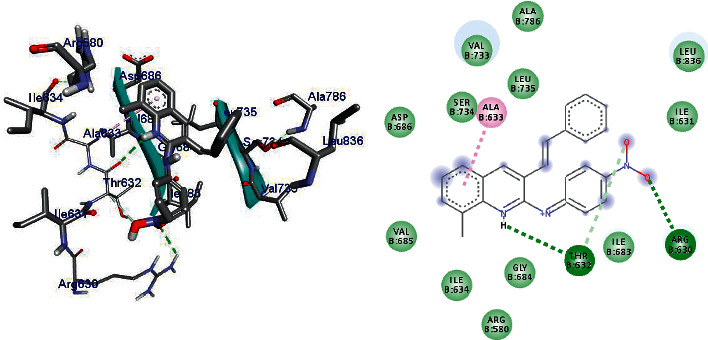
The binding interactions of compound 19 against *E.coli* DNA gyrase A (PDB ID: 1ZI0).

**Figure 6:**
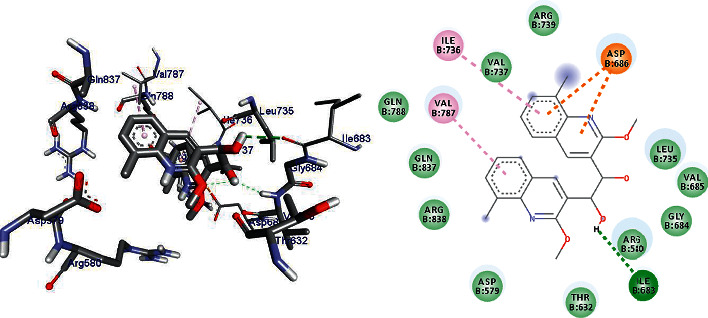
The binding interactions of compound 24 against *E.coli* DNA gyrase A (PDB ID: 1ZI0).

Compounds 14 (−6.7 kcal/mol), 16 (−6.7 kcal/mol), 19 (−6.9 kcal/mol), and 24 (−7.1 kcal/mol) showed good activities against *E.coli*, and among them, compound 24 revealed better activity [26]. Compounds **5** and **9** do not show any hydrogen bond interaction with any amino-acid residues within the active site. Compounds **5, 7, 9, 10,** and **22** docking results were partially matching the clinical drug ciprofloxacin interactions with amino-acid residues. Based on the in silico molecular docking analysis result, compound 24 (−7.1 kcal/mol) showed the highest binding affinity compared to ciprofloxacin (−7.3 kcal/mol). Therefore, compound 24 might be a better antibacterial agent than the other synthesized compounds reported herein. The binding affinity, H-bond, and residual interaction of nine compounds are summarized in Table 3, and the binding interactions of 19 and 24 against *E.coli* DNA gyrase A are depicted in Figures 2, 5, and 6.

In conclusion, quinolone-stilbenes-merged hybrid molecules were reported by utilizing Wittig reaction between 2-chloroquinoline-3-carbaldehyde derivatives and benzyltriphenylphosphonium chloride. The 2-chloroquinoline-3- carbldehyde derivatives were synthesized by Vilsmeier–Haack reaction, and the chlorine atom was replaced by various nucleophiles via a nucleophilic substitution reaction. A novel pinacol of quinoline was also synthesized by pinacolization of 2-methoxyquinoline-3-carbaldehyde with Al/KOH reagent system. The antibacterial activities of the compounds were evaluated with paper-disc diffusion against two Gram-negative and two Gram- positive bacterial strains with the best activity displayed by compound 19 against *E. coli* with an inhibition zone of 16.0 ± 0.82 mm and 14.67 ± 0.94 mm at 500 and 250 *μ*g/mL, respectively. This is close to ciprofloxacin which is used as a positive control. The results of *in silico* molecular docking evaluation of the compounds against *E. coli* DNA gyrase A were also in good agreement with *in vitro* antibacterial analysis. Two of the compounds, 19 and 24, exhibited the highest binding affinity comparable to ciprofloxacin. Some of these compounds (5, 14, 16, 19, and 24) possess potent antibacterial properties. The antioxidant properties of these compounds revealed that they were active only to a half extent of ascorbic acid at the same concentration. Finally, we recommended that both the antibacterial and antioxidant activities can be further optimized by incorporating active bioisostere groups on the phenyl ring of the hybrid scaffold.

## Figures and Tables

**Figure 4:**
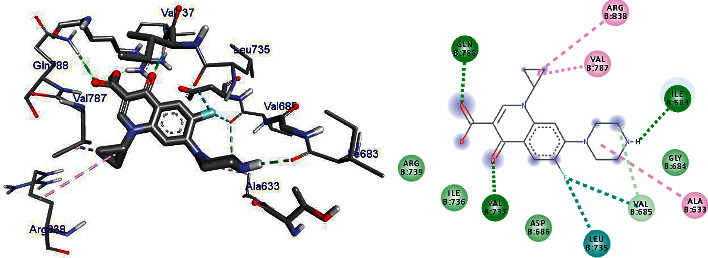
Percent radical scavenging activity of synthetic compounds.

**Table 1:** The synthesized compound and the synthetic procedure followed.

No.	Synthesized compound	Synthetic procedure
1	Acetanilide	An acetanilide was prepared by refluxing a mixture of aniline (25 mL) and acetic anhydride (25 mL) in a 250 mL round-bottom flask containing zinc powder (0.4 g) for an hour, and 200 mL crushed ice water was used to precipitate the yield. The yield was 23 g (62.3%); mp 112-113°C
2	2-Chloroquinoline-3-carbaldehyde (4)	Vilsmeier reagent was prepared by putting 29 mL (0.375 mol) of *N,N*-dimethylformamide in a 200 mL round-bottom flask, with a drying tube fitted to its neck, and it was cooled to 5°C using an ice bath. Then phosphorus oxychloride (98 mL, 1.05 mol)) was added to it dropwise from a dropping funnel while being stirred with a magnetic stirrer over a period of 30 min. Then, acetanilide (20.55 g, 0.15 mol) was added to the aforementioned mixture. After 5 minutes, the dropping funnel was replaced by an air condenser with a drying tube at its end, and it was heated at 90–95°C for 22 hours. The mixture was cooled to room temperature and poured into 400 mL crushed ice while being stirred mechanically. The precipitate was collected by suction filtration. The crude product was 17.5 g (61%)
3	(Z)-2-chloro-3-styrylquinoline (5)	A solution prepared by dissolving 0.4 g pellets of KOH in 1 mL water was added dropwise to a mixture of 2-chloroquinoline-3-carbaldehyde (0.5 g, 2.6 mmol) and benzyltriphenylphosphonium chloride (1.01 g, 2.6 mmol) dissolved in 10 mL DMF in a 100 mL round-bottom flask while being stirred with a magnetic stirrer. The stirring was continued for 5 hrs at which TLC analysis showed the complete consumption of 2-chloroquinoline-3-carbaldehyde. Then, the mixture was poured to 100 mL crushed ice water, and the precipitate was collected with suction filtration. The crude product was 0.62 g, 89.8%

**Table 2:** Continued table of the synthesized compounds and their synthetic procedure.

No	Synthesized compound	Synthetic procedure
5	(Z)-3-styrylquinolin-2(1H)-one (7)	Aqueous solution of KOH (1 mL, 40%) was added dropwise to a mixture of 2-oxo-1,2-dihydroquinoline-3-carbaldehyde (0.45 g, 2.6 mmol) and benzyltriphenylphosphonium chloride (1.01 g, 2.6 mmol) dissolved in 10 mL DMF in a 100 mL round-bottom flask while being stirred with a magnetic stirrer. After stirring for 4 hrs, the mixture was poured to 100 mL crushed ice water, and the precipitate was collected with suction filtration. The crude product was 0.55 g (85%)
6	2-Methoxyquinoline-3-carbaldehyde (8)	To the mixture of methanol (15 mL) and DMF (15 mL) in a 250 mL round-bottom flask were added chloroquinoline-3-carbaldehyde (1.0 g, 5.2 mmol) and potassium carbonate (1.5 g, 10.8 mmol). A water condenser was fitted to the flask, and the mixture was refluxed for 6 hours. The completion of the reaction was monitored by TLC, and the the excess methanol was removed by distillation. Then, the residue was added to 100 mL ice-cold water after being cooled to room temperature. The precipitate was collected by suction filtration and washed twice with 20 mL ice-cold water. The yield was a gray powder, 87 g (90%); mp 106–108°C

**Table 3:** Continued table of the synthesized compounds and their synthetic procedure.

No	Synthesized compound	Synthetic procedure
7	(Z)-2-methoxy-3-styrylquinoline (9)	An aqueous solution of KOH (1 mL, 40%) was added dropwise to a mixture of 2-methoxyquinoline-3-carbaldehyde (0.50 g, 2.6 mmol) and benzyltriphenylphosphonium chloride (1.01 g, 2.6 mmol) dissolved in 10 mL DMF in a 100 mL round-bottom flask while being stirred with a magnetic stirrer. The stirring was continued for 8 hrs at which TLC analysis showed the complete consumption of the reactant. Then, the mixture was poured into 100 mL crushed ice water, and the precipitate was collected with suction filtration. The crude product was 0.6 g, (88%); mp 138–140°C
8	2-(((2-((2-Hydroxyethyl)amino)quinolin-3-yl)methylene)amino)ethan-1-ol (10)	2-Chloroquiomoline-3-carbaldehyde (0.5 g, 2.6 mmol) was added to 10 mL 2-aminoethan-1-ol in a 100 mL round-bottom flask and heated to 90–95°C for an hour in a water bath. The completion of the reaction was monitored by TLC. The resulting mixture was cooled to room temperature and added to 100 mL cold ice water. The precipitate was separated by suction filtration and washed with 20 mL cold water. The product was a yellow powder, and the yield was 0.59 g (84%); mp 80–82°C
9	2-Chloro-8-methylquinoline-3-carbaldehyde (13)	The synthesis 2-chloro-8-methylquinoline-3-carbaldehyde was carried out with the procedure discussed above for the synthesis of 2-chloroquinoline-3-carbaldehyde, by replacing acetanilide with 2-methylacetanilide (21 g, 0.14 mol). The crude product was a yellow powder, and the yield was 17.94 g (62.3%)

**Table 4:** Continued table of the synthesized compounds and their synthetic procedure.

No	Synthesized compound	Synthetic procedure
10	(Z)-2-chloro-8-methyl-3-styrylquinoline (14)	1 mL 40% aqueous KOH was added dropwise to a mixture of 2-Chloro-8-methylquinoline-3-carbaldehyde (0.53 g, 2.6 mmol) and benzyltriphenylphosphonium chloride (1.01 g, 2.6 mmol) dissolved in DMF (10 mL) in 100 mL round bottom flask while being stirred with magnetic stirrer. The stirring was continued for 8 hrs at which TLC analysis showed the disappearance of the reactant. Then, the mixture was poured to 100 mL crushed ice water, and the precipitate was collected with suction filtration. The crude product was 0.64 g (88.2%)
11	8-Methyl-2-oxo-1,2-dihydroquinoline-3-carbaldehyde (15)	2-Chloro-8- methylquinoline-3-carbaldehyde (1.0 g, 5.3 mmol) was refluxed in a mixture of 6 M HCl (10 mL) and glacial acetic acid (15 mL) for 3 hrs with the progress of reaction followed by TLC. At end, the excess acetic acid was removed by distillation under reduced pressure. The residue was added to 50 mL crushed ice cold water, and the precipitate was collected by suction filtration, washed with cold water, and allowed to dry in a wood cupboard. The product was a yellow powder, and the yield was 0.79 g (80.2%); mp 176–178°C
12	(Z)-8-methyl-3-styrylquinolin-2(1H)-one (16)	Aqueous solution of KOH (1 mL, 40%) was added dropwise to a mixture of 8-methyl-2-oxo-1,2-dihydroquinoline-3-carbaldehyde (0.50 g, 2.6 mmol) and benzyltriphenylphosphonium chloride (1.01 g, 2.6 mmol) in DMF (10 mL) in a 100 mL round-bottom flask while being stirred with a magnetic stirrer. The stirring was continued for 7 hrs at which TLC analysis showed the complete consumption of the reactant. Then, the mixture was poured to 100 mL crushed ice water, and the precipitate was collected with suction filtration. The crude product was 0.55 g (85%)

**Table 5:** Continued table of the synthesized compounds and their synthetic procedure.

No	Synthesized compound	Synthetic procedure
13	8-Methyl-2-((4- nitrophenyl)amino)quinoline-3-carbaldehyde (18)	Compound 18 was synthesized by mixing *p*-nitroaniline (0.34 g, 0.0024 mol), 2-chloro-8-methylquinoline-3-carbaldehyde (0.5 g, 0.0024 mol), potassium carbonate (0.67 g, 0.0047 mol), and moist copper powder (0.1 g, 0.0016) in a 100 mL round-bottom flask containing *N,N*-dimethylformamide (20 mL). A water condenser was fitted into the neck, and the mixture was refluxed for 5 hours; meanwhile, the progress of the reaction was attended by TLC. After being cooled to room temperature, it was added to 100 mL crushed ice water. The solid product was separated by suction filtration and washed with 5% HCl solution (50 mL) and allowed to dry in air. The product was a gray powder, and the yield was 0.93 g (85.3%)
14	8-Methyl-N-(4-nitrophenyl)-3-styrylquinolin-2-amine (19)	An aqueous solution of KOH (1 mL, 40%) was added dropwise to a mixture of 8-methyl-2-((4-nitrophenyl)amino)quinoline-3-carbaldehyde (0.68 g, 2.2 mmol) and benzyltriphenylphosphonium chloride (0.86 g, 2.2 mmol) dissolved in DMF (15 mL) in a 100 mL round-bottom flask while being stirred with a magnetic stirrer. The stirring was continued overnight until TLC analysis showed the complete conversion of the reactant. Then, the mixture was poured to 150 mL crushed ice water, and the precipitate was separated by suction filtration and washed with 30 mL cold water. The product was a pink powder, and the crude yield was 1.07 g (92%)

**Table 6:** Continued table of the synthesized compounds and their synthetic procedure.

No	Synthesized compound	Synthetic procedure
15	(4-((3-Formyl-8-methylquinolin-2-yl)methyl)phenyl)acetamide (21)	N-(4-hydroxyphenyl)acetamide (0.36 g, 2.4 mmol)), 2-chloro-8-methylquinoline-3-carbaldehyde (0.5 g, 2.4 mmol), potassium carbonate (0.67 g, 4.7 mmol), and 0.1 g moist copper powder were mixed to a 100 mL round-bottom flask in DMF (20 mL). An air condenser was fitted to the neck and refluxed for 5 hours until N-(4-hydroxyphenyl)acetamide completely disappeared on TLC analysis. Then, it was cooled to room temperature and poured to 100 mL crushed ice water. The precipitate was separated by suction filtration and washed with of 5% aqueous NAOH (50 mL) and air dried. The product was a gray powder, and the yield was 1.0 g (89%)
16	(Z)-N-(4-((8-methyl-3-styrylquinolin-2-yl)methyl)phenyl)acetamide (22)	An aqueous solution of KOH (1 mL, 40%) was added dropwise to a mixture of N-(4-((3-formyl-8-methylquinolin-2-yl)methyl)phenyl)acetamide (0.94 g, 2.0 mmol) and benzyltriphenylphosphonium chloride (0.78 g, 2.0 mmol) dissolved in DMF (15 mL) in a 100 mL round-bottom flask while being stirred with a magnetic stirrer. The stirring was continued for 16 hours at which TLC analysis showed the complete conversion of the reactant. Then, the mixture was poured to 150 mL crushed ice water, and the precipitate was separated by suction filtration and washed with 30 mL cold water. The crude product was 0.99 g (91.1%)
17	Methoxy-5-methylquinolin-3-yl)-2-(2-methoxy-8-methylquinolin-3-yl)ethane- 1,2-diol (**24**)	2-Methoxy-8-methylquinoline-3-carbaldehyde (0.6 g, 3 mmol)) aluminum powder (0.16 g, 6 mmol) and potassium hydroxide (1.1 g, 18 mmol) were added to 10 mL methanol in a 100 ml round-bottom flask with an air condenser. The flask was mounted over a magnetic stirrer, and the reaction mixture was stirred for 12 hours. The progress of the reaction was monitored by TLC. The reaction mixture was filtered to remove unreacted aluminum powder, and 50 mL water was added to the filtrate. The precipitate was collected by suction filtration and air dried. The crude product was 0.53 g (43.7%)

**Table 7:** The *Z*-factor of the antibacterial assay of the synthetic compounds and ciprofloxacin.

Bacterial strains	*E. coil*		*S. typhimurium*		*S. aureus*		*B. subtilis*	
Concentration in *μ*g/mL	250	500	250	500	250	500	250	500
Compounds								
5	0.52	0.45	No	No	No	No	No	No
9	0.66	0.66	No	No	No	No	No	No
10	0.41	0.61	No	No	No	No	No	No
14	0.3	0.74	0.06	0.34	No	No	No	No
16	No	No	−1.0	−0.6	No	No	No	No
19	−0.06	−0.45	No	No	No	No	No	No
24	No	No	0.86	0.21	−0.46	−0.46	No	No
Ciprofloxacin	0.92	0.92	0.82	0.82	0.82	0.88	0.83	0.84

No = inhibition zone was not observed; ciprofloxacin was used as positive control.

**Table 8:** Antibacterial activity of the synthetic compounds.

Bacterial strains		*S. aureus*		*B. subtilis*		*E. coli*		*S. typhimurium*	
Concentration (*μ*g/mL)		500	250	500	250	500	250	500	250
Compound no.	5	No	No	No	No	10 ± 0.82^Aa^	8.67 ± 1.25^Ab^	8.33 ± 1.72^D^	6.67 ± 0.47^D^
	7	No	No	No	No	No	No	No	No
	9	No	No	No	No	8.0 ± 1.25^Ba^	8.0 ± 1.25^Bb^	No	No
	10	No	No	No	No	9.33 ± 1.25^a^	8.0 ± 0.82b	No	No
	14	No	No	No	No	12.67 ± 0.82^AB^	11.33 ± 1.70^AB^	No	No
	16	No	No	No	No	No	No	9.67 ± 1.25^D^	9.67 ± 0.47^De^
	19	No	No	No	No	16.0 ± 0.82^AB^	14.67 ± 0.94^AB^	No	No
	22	No	No	No	No	No	No	No	No
	24	16.0 ± 1.63^C^	13.67 ± 0.47^C^	No	No	No	No	8.67 ± 0.47^D^	8.33 ± 1.25^De^
	Ciprofloxacin	11.67 ± 0.47^C^	11.33 ± 0.67^C^	15.59 ± 0.82	14.67 ± 0.82	18.67 ± 0.47^AB^	18.67 ± 0.47^AB^	13.56 ± 0.82^D^	11.33 ± 0.67^D^

No = inhibition zone was not observed; all results are expressed as mean ± SD of triplicate experiments; means with the same letter (upper case) within the column are significantly different; means with the same letter (lower case)) in the same column are not significantly different.

**Table 9:** Percent radical scavenging activity and IC50 values of synthetic compounds.

Compounds	% radical scavenging activity at				IC50 (*μ*g/ml)
	12.5 *μ*g/mL	25 *μ*g/mL	50 *μ*g/mL	1005 *μ*g/mL	
5	32.21 ± 0.44	35.39 ± 0.67	41.06 ± 0.87	50.10 ± 0.25	43.32
7	40.09 ± 0.47	42.21 ± 0.82	43.27 ± 0.47	45.93 ± 0.67	33.08
9	33.72 ± 0.27	42.48 ± 0.52	43.81 ± 0.47	43.98 ± 0.67	41.58
10	33.54 ± 0.82	36.73 ± 0.82	38.58 ± 0.67	40.09 ± 0.92	37.48
14	20.97 ± 0.82	34.07 ± 0.82	36.64 ± 1.25	36.73 ± 1.25	33.41
16	27.88 ± 0.67	31.86 ± 0.72	38.50 ± 0.47	41.06 ± 0.87	35.34
19	31.15 ± 0.82	35.13 ± 0.82	45.13 ± 0.67	55.54 ± 0.92	42.63
22	37.17 ± 0.57	39.555 ± 0.87	48.58 ± 0.67	52.21 ± 0.72	45.03
24	33.62 ± 0.47	36.73 ± 0.67	42.21 ± 1.25	42.21 ± 0.82	39.06
Ascorbic acid	86.9 ± 0.52	89.99 ± 0.67	90.03 ± 0.71	90.33 ± 0.82	4.50

The antioxidant activity of the synthetic compounds was also elaborated in Figure 4 with a line diagram.

**Table 10:** The *Z*-factor of radical scavenging activity of synthetic compounds and ascorbic acid.

Compounds	Concentration in *μ*g/mL			
	12.5	25	50	100
5	0.95	0.93	0.90	0.92
7	0.94	0.91	0.92	0.90
9	0.96	0.92	0.92	0.90
10	0.92	0.93	0.92	0.90
14	0.94	0.92	0.89	0.88
16	0.94	0.93	0.93	0.99
19	0.93	0.92	0.91	0.85
22	0.93	0.91	0.90	0.88
24	0.94	0.92	0.88	0.90
Ascorbic acid	0.98	0.98	0.98	0.97

**Table 11:** Molecular docking results of synthesized compounds against *E.coli* DNA gyrase A (PDB ID 1ZI0).

S. no.	Ligan ds	Affinity (kcal/mol)	H-bond	Residual amino-acid interactions	
				Hydrophobic/Pi-cation/Pi-anion/Pi-alkyl interactions	van der Waals interactions
1	5	−6.1	—	Ile-736, Asp-686, Val-787	Val-685, Val-737, Ile-683, Gly-684, Thr-632, Gln-788
2	7	−6.4	Val-737	Ile-736, Arg-739	Val-685, Leu-735, Gln-788
3	9	−6.2	—	Ile-736, Asp-686	Val-685, Val-737, Ile-683, Gly-684, Thr-632, Val-787, Gln-788, Leu-735
4	10	−5.6	Ile-683, Leu-735	Le-736	Gly-684, Val-685, Gln-788, Ser-734, Val-787
5	14	−6.7	Arg-838	Ile-736, Val-787, gln-837	Val-685, Asp-686, Leu-735, Val-737, Ala-786, Gln-788, Leu-836
6	16	−6.7	Leu-735	Val-787	Val-685, Asp-686, Ser-734, Val-737, Ile-683, Gly-684, Arg-838, Leu-836, Gln-837
**7**	**19**	−6.9	Arg-630, Thr-632	Ala-633	Arg-580, Ile-634, Ile-631, Ile-683, Gly-684, Val-685, Asp-686, Val-733, Ser-734, Leu-735, Ala-786, Leu-836
**8**	**22**	−6.5	Ile-634, Arg-580	Ala-633, Ile-736, Asp-686	Asp-579, Thr-632, Ile-683, Gly-684, Val-685, Val-737, Leu-735, Gln-788
**9**	**24**	−7.1	Ile-683	Val-787, Ile-736, Asp-686	Arg-580, Thr-632, Asp-579, Gly-684, Val-685, Leu-735, Val-737, Arg-739, Gln-788, Gln-837, Arg-838
Ciprofloxacin		−7.3	Ile-683, Val-737	Ala-633, Val-685, Leu-735, Val-787, Arg-838	Gly-684, Asp-686, Ile-736, Arg-739

## Data Availability

The data supporting the results are available from the corresponding author.

## References

[1] Bergogne-Bérézin E., Towner K. J. (1996). Acinetobacter spp. as nosocomial pathogens: microbiological, clinical, and epidemiological features. *Clinical Microbiology Reviews*.

[2] Mishra S. K., Rijal B. P., Pokhrel B. M. (2013). Emerging threat of multidrug resistant bugs—Acinetobacter calcoaceticus baumannii complex and Methicillin resistant Staphylococcus aureus. *BMC Research Notes*.

[3] Desai N. C., Patel B. Y., Dave B. P. (2017). Synthesis and antimicrobial activity of novel quinoline derivatives bearing pyrazoline and pyridine analogues. *Medicinal Chemistry Research*.

[4] Verbanac D., Malik R., Chand M. (2016). Synthesis and evaluation of antibacterial and antioxidant activity of novel 2-phenyl-quinoline analogs derivatized at position 4 with aromatically substituted 4H-1,2,4-triazoles. *Journal of Enzyme Inhibition and Medicinal Chemistry*.

[5] Musiol R., Jampilek J., Kralova K. (2007). Investigating biological activity spectrum for novel quinoline analogues. *Bioorganic & Medicinal Chemistry*.

[6] Van de Walle T., Boone M., Van Puyvelde J. (2020). Synthesis and biological evaluation of novel quinoline-piperidine scaffolds as antiplasmodium agents. *European Journal of Medicinal Chemistry*.

[7] Rashad A. E., El-Sayed W. A., Mohamed A. M., Ali M. M. (2010). Synthesis of new quinoline derivatives as inhibitors of human tumor cells growth. *Archiv der Pharmazie*.

[8] Shobeiri N., Rashedi M., Mosaffa F. (2016). Synthesis and biological evaluation of quinoline analogues of flavones as potential anticancer agents and tubulin polymerization inhibitors. *European Journal of Medicinal Chemistry*.

[9] Zhang L., Sun F., Li Y. (2007). Rapid synthesis of iminosugar derivatives for cell-based in situ screening: discovery of “hit” compounds with anticancer activity. *ChemMedChem*.

[10] Lam K.-H., Gambari R., Lee K. K.-H. (2014). Preparation of 8-hydroxyquinoline derivatives as potential antibiotics against Staphylococcus aureus. *Bioorganic & Medicinal Chemistry Letters*.

[11] Orhan Puskullu M., Tekiner B., Suzen S. (2013). Recent studies of antioxidant quinoline derivatives. *Mini-Reviews in Medicinal Chemistry*.

[12] Marble D. A., Bosso J. (1989). Norfloxacin: a quinoline antibiotic. *Annals of Pharmacotherapy*.

[13] Rizk O. H., Mahran M. A., El-Khawass S. M., Shams El-Dine S. A., Ibrahim E.-S. A. (2005). Synthesis of some new antimicrobial thiadiazolyl and oxadiazolyl quinoline derivatives. *Medicinal Chemistry Research*.

[14] Yağci R. (2007). Penetration of second-, third-, and fourth-generation topical fluoroquinolone into aqueous and vitreous humour in a rabbit endophthalmitis model. *Eye*.

[15] Xiao Z.-P., Wang X.-D., Wang P.-F. (2014). Design, synthesis, and evaluation of novel fluoroquinolone-flavonoid hybrids as potent antibiotics against drug-resistant microorganisms. *European Journal of Medicinal Chemistry*.

[16] (2003). Stilbenes synthesis. *Journal of Medicinal Chemistry*.

[17] Wyrzykiewicz E., Wendzonka M., Kędzia B. (2006). Synthesis and antimicrobial activity of new (E)-4-[piperidino (4′-methylpiperidino-, morpholino-) N-alkoxy]stilbenes. *European Journal of Medicinal Chemistry*.

[18] Chalal M., Klinguer A., Echairi A., Meunier P., Vervandier-Fasseur D., Adrian M. (2014). Antimicrobial activity of resveratrol analogues. *Molecules*.

[19] Miliovsky M., Svinyarov I., Mitrev Y. (2013). A novel one-pot synthesis and preliminary biological activity evaluation of cis-restricted polyhydroxy stilbenes incorporating protocatechuic acid and cinnamic acid fragments. *European Journal of Medicinal Chemistry*.

[20] Sun H.-Y., Xiao C.-F., Cai Y.-C. (2010). Efficient synthesis of natural polyphenolic stilbenes: resveratrol, piceatannol and oxyresveratrol. *Chemical & Pharmaceutical Bulletin*.

[21] Meth-Cohn O., Narine B., Tarnowski B. (1981). A versatile new synthesis of quinolines and related fused pyridines, part 5. The synthesis of 2-chloroquinoline-3-carbaldehydes. *Journal of the Chemical Society, Perkin Transactions*.

[22] Zeleke D., Eswaramoorthy R., Belay Z., Melaku Y. (2020). Synthesis and antibacterial, antioxidant, and molecular docking analysis of some novel quinoline derivatives. *Journal of Chemistry*.

[23] Brenner D. J., Staley J. T., Krieg N. R. (1994). *Bergey’s Manual® of Systematic Bacteriology Classification of Procaryotic Organisms and the Concept of Bacterial Speciation*.

[24] Narramore S., Stevenson C. E. M., Maxwell A., Lawson D. M., Fishwick C. W. G. (2019). New insights into the binding mode of pyridine-3-carboxamide inhibitors of E. coli DNA gyrase. *Bioorganic & Medicinal Chemistry*.

[25] Viegas-junior C., Danuello A., Bolzani S., Barreiro E. J., Alberto C., Fraga M. (2007). Molecular hybridization: a useful tool in the design of new drug prototypes. *Current Medical Chemistry*.

[26] Jarwal N., Thankachan P. P. (2015). Theoretical study of the Wittig reaction of cyclic ketones with phosphorus ylide. *Journal of Molecular Modeling*.

[27] Bergelson L. D., Barsukov L. I., Shemyakin M. M. (1967). The stereochemistry of the Wittig reaction with non-stabilized and semistabilized ylids. *Tetrahedron*.

[28] Gomberg M., Bachmann W. E. (1927). The reducing action of a mixture of magnesium iodide (or bromide) and magnesium on aromatic ketones. Probable formation of magnesium subiodide (or subbromide). *Journal of the American Chemical Society*.

[29] Khurana J. M., Sehgal A., Gogia A., Manian A., Maikap G. C. (1996). Pinacolization and reduction of aromatic carbonyls with aluminium-KOH. *Journal of the Chemical Society, Perkin Transactions 1*.

[30] Sotto N., Billamboz M., Chevrin-Villette C., Len C. (2015). Selective pinacol coupling on regeneratable supported acids in sole water. *The Journal of Organic Chemistry*.

[31] Khurana J. M., Sehgal A. (1994). Rapid pinacolization of carbonyl compounds with aluminium-KOH. *Journal of the Chemical Society, Chemical Communications*.

[32] Yuan S.-Z., Wang Z.-Y., Li Z. (2006). Reduction and coupling reaction of carbonyl compounds by aluminum powder and a small amount of oxalic acid in water. *Chinese Journal of Chemistry*.

[33] Zhang J.-H., Chung T. D. Y., Oldenburg K. R. (1999). A simple statistical parameter for use in evaluation and validation of high throughput screening assays. *Journal of Biomolecular Screening*.

[34] Liang N., Kitts D. (2014). Antioxidant property of coffee components: assessment of methods that define mechanisms of action. *Molecules*.

[35] Bukhari S. B., Memon S., Tahir M. M., Bhanger M. I. (2008). Synthesis , characterization and investigation of antioxidant activity of cobalt—quercetin complex. *Journal of Molecular Structure*.

[36] Puskullu M. O., Shirinzadeh H., Nenni M., Gurer-Orhan H., Suzen S. (2016). Synthesis and evaluation of antioxidant activity of new quinoline-2-carbaldehyde hydrazone derivatives: bioisosteric melatonin analogues. *Journal of Enzyme Inhibition and Medicinal Chemistry*.

[37] Ziraldo R., Hanke A., Levene S. D. (2019). Kinetic pathways of topology simplification by type-II topoisomerases in knotted supercoiled DNA. *Nucleic Acids Research*.

